# How does Epstein–Barr virus (EBV) complement the activation of *Myc* in the pathogenesis of Burkitt's lymphoma?

**DOI:** 10.1016/j.semcancer.2009.07.007

**Published:** 2009-12

**Authors:** Martin J. Allday

**Affiliations:** Department of Virology, Faculty of Medicine, Imperial College London, Norfolk Place, London W2 1PG, UK

**Keywords:** EBV, Burkitt's lymphoma, *Myc*, Apoptosis, Senescence

## Abstract

A defining characteristic of the aggressive B cell tumour Burkitt's lymphoma (BL) is a reciprocal chromosomal translocation that activates the *Myc* oncogene by juxtaposing it to one of the immunoglobulin gene loci. The consequences of activating *Myc* include cell growth and proliferation that can lead to lymphomagenesis; however, as part of a fail-safe mechanism that has evolved in metazoans to reduce the likelihood of neoplastic disease, activated oncogenes such as *Myc* may also induce cell death by apoptosis and/or an irreversible block to proliferation called senescence. For lymphoma to develop it is necessary that these latter processes are repressed. More than 95% of a subset of BL – known as endemic (e)BL because they are largely restricted to regions of equatorial Africa and similar geographical regions – carry latent Epstein–Barr virus (EBV) in the form of nuclear extra-chromosomal episomes. Although EBV is not generally regarded as a driving force of BL cell proliferation, it plays an important role in the pathogenesis of eBL. Latency-associated EBV gene products can inhibit a variety of pathways that lead to apoptosis and senescence; therefore EBV probably counteracts the proliferation-restricting activities of deregulated *Myc* and so facilitates the development of BL.

## 1 Burkitt's lymphoma

Burkitt's lymphoma (BL) is a high grade, non-Hodgkin's B cell tumour. Endemic BL (eBL) generally affects children and occasionally young adults in tropical Africa and a few other geographically defined areas such as Papua New Guinea. These are equatorial regions where malaria is hyperendemic and co-infection with malaria parasites seems to be an important co-factor in the development of eBL (reviewed in [1–3] and elsewhere in this volume). Up to 98% of eBL carry EBV and in the majority of cases only the viral episome-maintenance protein EBNA1 and the non-coding EBER RNAs are expressed in the tumour cells (see later). Because of the cellular phenotype and mutations found in immunoglobulin variable gene sequences, BL is generally believed to originate from germinal centre or post-germinal centre B cells [4] and reviewed in [5]. However, the discovery during normal EBV persistence of dividing memory B cells expressing the EBNA1 (and EBERs) latency programme seen in eBL has raised the possibility that EBV-positive BL may be derived from memory B cells that cannot exit from the cell cycle ([3,6–8]; for a fuller discussion see later sections of this article).

Although the incidence of BL is highest in equatorial regions, it occurs worldwide at a much lower prevalence. In contrast to eBL, these sporadic cases of BL (sBL) are only occasionally associated with EBV. For instance in the USA and Western Europe only about 15% of cases harbour the EBV genome. The exception here is when BL occurs in individuals with HIV/AIDS. In AIDS patients the incidence of BL is high and about 40–50% of these are EBV-positive [9]. Regardless of their origin and EBV status, eBL and sBL are histologically indistinguishable; furthermore the molecular hallmark of every BL is a cytogenetic abnormality that deregulates the *Myc* oncogene. Cell growth and proliferation in BL is driven by the constitutively active *Myc*, and although the end result and major cellular pathways that are disrupted are essentially similar in sBL and eBL, we will see that the molecular details of pathogenesis differ somewhat because of the contribution of EBV latency gene products in the latter.

## 2 *Myc* activation is a defining characteristic of BL

A reciprocal chromosomal translocation between the *Myc* proto-oncogene and one of three immunoglobulin genes (*Ig*/*Myc*) is a hallmark of BL, irrespective of whether or not they carry EBV or their geographical origin (reviewed in [1,10,11] and elsewhere in this volume). The three most common translocations involve a breakpoint in the long arm of chromosome 8 at the 8q24 locus, adjacent to or within the *Myc* gene. The most frequent translocation – found in 80% of cases – transposes the telomeric region of chromosome 8 to the immunoglobulin heavy chain gene (*IgH*) on chromosome 14 [t(8:14)]. The remainder involve either the kappa [t(2:8] or lambda [t(8:22)] immunoglobulin light chain genes (*IgL*). For reasons that remain unknown, in the t(8:14) translocations associated with EBV-carrying endemic BL, the breakpoint in *Myc* typically occurs more than 100 kb upstream of the first coding exon and the *Ig* breakpoint occurs in the VDJ region of the *IgH* gene. In contrast the breakpoints in sporadic and many AIDS–BL generally occur between exons 1 and 2 of *Myc* and the *IgH* class switch (S) region.

Since in most cases of BL the DNA breakpoints are found in rearranged VJ regions or in S regions of the *IgH* loci, it is now generally accepted that these chromosomal translocations are mediated by aberrant somatic hypermutation (SHM) or class switch recombination (CSR) activity, occur in activated germinal centre (GC) B cells and require the DNA-modifying enzyme known as activation-induced deaminase (AID). AID is highly expressed in GCs where it catalyses deamination of cytidine residues, resulting in U:G mismatches that are processed to produce double strand (ds) DNA breaks. These ds breaks are essential for CSR, but also occur in SHM. There is now overwhelming evidence that activated AID can directly mediate the translocation of *Myc* in GC B cells and because the J_H_ region is preferentially affected and there are often signs of antigen selection in eBL, this has led to the attractive hypothesis that in EBV-positive BL translocation occurs as a consequence of aberrant SHM in late GC B cells [11–17].

An outcome of *Ig*/*Myc* translocation is that the proto-oncogene is brought under the control of a transcriptionally active *Ig* locus, leading to deregulated constitutive expression of the translocated gene. In this context *Myc* becomes an active oncogene. As well as facilitating chromosomal translocations, SHM can contribute to lymphomagenesis further by mutating non-*Ig* genes; these may include the translocated *Myc* genes that can accumulate mutations in the region associated with breakpoints and often in the Myc transactivation domain (for example [18]).

## 3 The consequences of *Myc* activation

As a result of translocation into an *Ig* locus in a mature B cell, *Myc* RNA is constitutively expressed and Myc protein accumulates to higher levels than are seen in any normal B cells. This is due partly to the increased transcription and also in some cases because mutations that introduce single amino acid substitutions (e.g. Thr58Ala) lead to stabilization of Myc protein because of reduced susceptibility to proteasome-mediated turn-over [19,20]. Myc is a sequence-specific DNA-binding transcription factor and several high-throughput screens indicate that the Myc target-gene network corresponds to about 15% of all known genes [21]. In B cells Myc appears to be particularly important, acting as a major transcriptional hub that links a hierarchy of multiple sub-hubs directly or indirectly regulating the transcription of a vast number of genes [22,23].

**Fig. 1:**
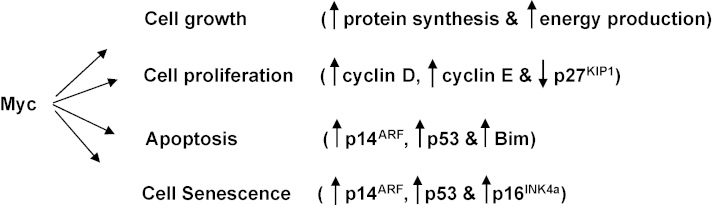
Pro- and anti-proliferative consequences of deregulating the proto-oncogene *Myc*. Increased protein synthesis and energy production and the resulting increase in cell size lead to cell division. Proliferation is enhanced by specific induction of cyclins D and E, and the repression of the cyclin-dependent kinase inhibitor p27^KIP1^. Apoptosis is induced by at least two pathways: either the induction of p14^ARF^ leading to an increase in p53 or the induction of Bim. Senescence may also be initiated via the induction of p14^ARF^/p53 and also the induction of the cdki p16^INK4a^. For references see text. Vertical arrows indicate increases or decreases.

Targets of Myc include groups of genes regulating cell cycle progression, cell growth (including metabolism, ribosome biogenesis, protein synthesis and energy production) apoptosis and senescence ([21,24–26]; Fig. 1). Stimulation of global protein synthesis by Myc directly augments cell size and this alone is probably sufficient to initiate cell cycle progression [27,28]. This induction of global protein synthesis and accompanying alteration in translational control appears to be a rate-limiting determinant of cancer initiation by Myc. Moreover it was found in murine B cells that activated *Myc* induces supernumerary centrosomes and genomic instability that is also dependent on the deregulation of protein synthesis [28]. Evidently the aberrant induction of cell growth is central to the role of Myc as an oncoprotein. Further insight into Myc's oncogenic potency is also emerging with the demonstration that it regulates and is regulated by a network of micro-RNAs (miRNAs) involved in the modulation of tumorigenesis [29–33].

The net effect of deregulating and over-expressing Myc is cell growth, uncontrolled proliferation but also a reduced threshold for the induction of apoptosis. *Myc* translocation in mature B cells is therefore accompanied by rapid mitogen-independent cell division, but a concomitant increase in the rate of cell death. Consistent with this, the histological appearance of BL and the behaviour of explanted cells in culture both indicate that BL cells have a high proliferative index but are very prone to apoptosis [1].

## 4 Myc-induced apoptosis—a central role for p53

The seminal discovery that Myc can actually trigger rapid apoptosis (as well as proliferation), led to the hypothesis that apoptotic pathways must be disabled for oncogenes to promote cell transformation and cancer [24,34,35]. This in turn led to the concepts of ‘oncogenic stress’ and ‘intrinsic tumour suppression’ and descriptions of fail-safe mechanisms that prevent deregulated oncogenes inducing neoplastic disease [36,37]. Mouse models that were established to mimic the translocation of *Myc* in B cells and its role in lymphomagenesis have been very fruitful in revealing the nature of Myc-responsive fail-safe mechanisms. Deregulated expression of *Myc* in B cells – activated by placing it under the control the *IgH* enhancer (*Eμ-Myc*) – induces the accumulation and activation of the tumour suppressor p53. This is largely because Myc stimulates, via E2F transcription factors, expression of the gene in the *CDKN2A* locus known as *p19*^*ARF*^ in mice and *p14*^*ARF*^ in humans. The ARF proteins interact with and antagonise the negative regulator of p53 called MDM2 [38–40]. Apoptosis is then induced primarily by p53 accumulating and transactivating genes that encode pro-apoptotic proteins such as Bax, Bid, Noxa and Puma rather than genes such as *p21*^*WAF1*^ that induce cell cycle arrest [41,42]. Additionally, p53 may directly activate the apoptotic machinery by initiating the release of cytochrome c from mitochondria [43].

In mice carrying an *Eμ*-*Myc* transgene, most clonal B lymphomas arise with either p53-mutations or *p19*^*ARF*^ deletions or they over-express MDM2. In BL it has been reported that about 30% of BL and up to 70% of BL-derived cell lines carry p53-mutations. Moreover, those BL with wild type p53 sometimes over-express MDM2 or lose *p14*^*ARF*^ expression by homozygous gene deletion or promoter methylation [44]. It seems that many, but not all, Myc-driven lymphomas acquire genetic or epigenetic changes that compromise the ARF/MDM2/p53 axis and so avoid Myc-induced apoptosis.

## 5 The special relationship between Myc and Bim

The ‘intrinsic’ or ‘mitochondrial’ apoptosis pathway is initiated by BH3 (Bcl-2 homology domain 3)-only proteins (including Bad, Bid, Bik, Bim, Noxa and Puma); these are a pro-apoptotic sub-group of the Bcl-2-family. To kill cells they all require the action of the Bax/Bak pro-apoptotic family members and the ‘intrinsic/mitochondrial’ pathway is regulated by the inhibitory action of Bcl-2 (the prototype) and other anti-apoptotic members of the Bcl-2-family, for example Bcl-X_L_ and Bfl-1 [45].

The pro-apoptotic family member Bim [Bcl-2-interacting mediator of cell death also known as Bcl-2-like protein 11 (BCL2L11)] acts as a potent, direct initiator of apoptosis because it binds with high affinity to Bcl-2 and all the other pro-survival family members to inactivate them. Bim also binds and activates pro-apoptotic Bax to initiate cytochrome c release from mitochondria [45,46]. Bim is particularly important in the immune system, acting as a major regulator of life-and-death decisions during lymphocyte development including the negative selection of auto-reactive B cells and developmentally programmed death of low-affinity antibody-expressing germinal centre-derived B cells [45,47,48]. *Bim* −/− mice accumulate excess lymphoid and myeloid cells and loss of Bim accelerates B-lymphomagenesis induced by an *Eμ*-*Myc* transgene. Even loss of a single allele of *Bim* accelerates lymphomagenesis significantly, indicating *Bim* is a haploinsufficient tumour suppressor and that the level of Bim protein is a critical rate-limiting factor in B cell survival [49]. Consistent with this, recent studies have revealed deletions or methylation of the *Bim* locus in a variety of human B lymphomas ([50,51] and our unpublished observations).

In *Eμ*-*Myc* mice with normal *Bim*, B lymphomas arise with relatively low penetrance (often over more than a year) and in most tumours the p19^ARF^/MDM2/p53 pro-apoptotic tumour-suppressor pathway has been inactivated (usually by mutation of p53 or p19^ARF^). However, in *Bim* −/− *Eμ-Myc* mice, tumours arise much more rapidly and the selection pressure for functional inactivation of the p53 apoptotic pathway appears to be removed [49]. Extending this mouse model of *Myc*-induced lymphoma to human lymphomagenesis, Lowe and colleagues revealed a very specific relationship between *Myc* and *Bim* in the pathogenesis of BL [52]. As we have seen, deregulated *Myc* is a hallmark of all BL and sometimes point mutations, probably arising as the result of aberrant AID during SHM, are found in the translocated *Myc* alleles. These mutations have a tendency to cluster in conserved box 1 (residues 44–65). Two common mutants of Myc that are found in BL – Pro57Ser and Thr58Ala – retain their ability to stimulate proliferation and activate *p14*^*ARF*^ and p53, but are defective at promoting apoptosis. This is because – unlike wild type Myc – they fail to induce the expression of Bim when over-expressed [52]. Since tumours carrying codon 57 or 58 mutations of *Myc* often retain a functional p53 pathway, but wild type *Myc* is generally accompanied by genetic lesions in the p53 pathway, it has been proposed that *Myc*-induced apoptosis is dependent on a cell tipping over a delicately balanced death threshold. Survival therefore involves inactivation of any one of several Myc effectors (e.g. p14/p19^ARF^, p53 or Bim) to cause apoptosis-firing to drop below the critical threshold and allow cell proliferation and the development of a tumour [37,52,53].

## 6 Myc, senescence and the *CDKN2A* locus

Oncogenic stress initiated by the inappropriate activation of oncogenes not only triggers apoptosis, but also, in certain circumstances, initiates a cell cycle arrest and a phenotype resembling that of aged, senescent cells. This is known as premature senescence and is generally characterised by an increase in the levels of several effectors of arrest that include p14^ARF^, p53, p21^WAF1^ and p16^INK4a^[54,55]. Although the responses that focus on the *CDKN2A* locus (that encodes both p14^ARF^ and p16^INK4a^ as the result of alternative splicing and reading frames) are generally associated with aberrant signalling from the oncoproteins Ras and Raf, Myc also targets this locus. As we have seen, the response to Myc is most commonly the initiation of apoptosis via p14^ARF^ and p53, but there is some evidence that Myc can also up-regulate *p16*^*INK4a*^ in human cells [25]. The molecular circuitry is probably very complex and may involve the repressor of transcription and Polycomb group (PcG) protein Bmi-1. Myc operationally cooperates with Bmi-1 in the induction of mouse lymphomas and the simplest interpretation is that while Myc activates the *CDKN2A* locus inducing apoptosis or arrest, Bmi-1 in a polycomb complex mediates repression of the locus via targeted epigenetic histone modifications and so the balance is shifted in favour of survival and proliferation [25,37].

It is clear why activation of *Myc* in lymphomas would be accompanied by genetic or epigenetic lesions that suppress apoptosis, but the link between Myc and senescence in B cells is less apparent. However, since the *p16*^*INK4a*^ promoter commonly becomes methylated at CpG dinucleotides in BL, this indicates that suppression of the growth arrest/senescence pathway is probably important in the pathogenesis of BL [44,56].

## 7 EBV latency

**Fig. 2:**
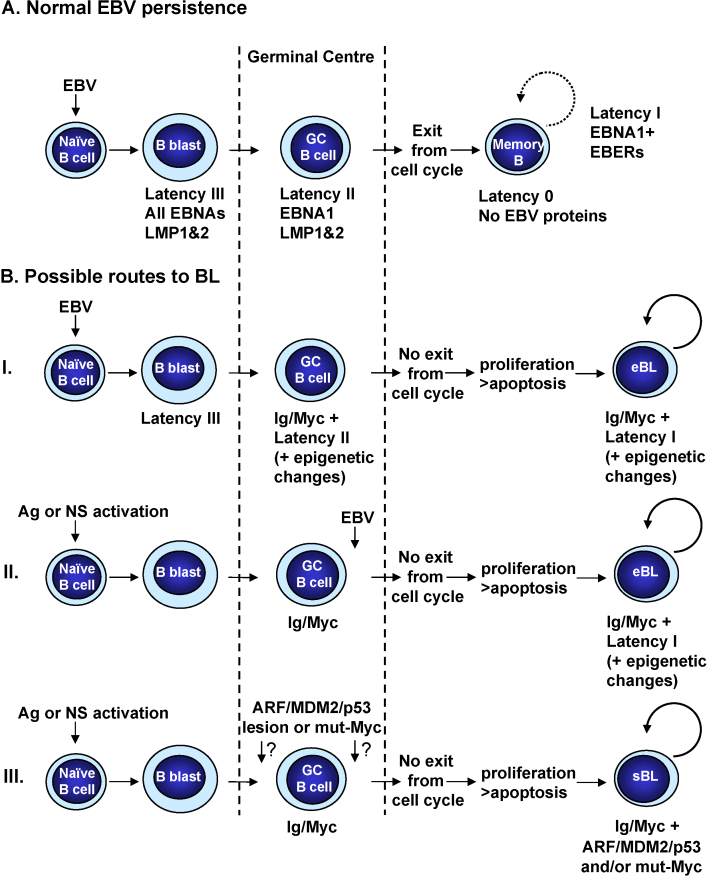
(A) EBV persistence. *In vivo*, EBV is thought to utilize normal B cell development and differentiation pathways to gain access to its site of long-term persistence, the memory B cell. The schematic is based on the model proposed by Thorley-Lawson and colleagues and is described more fully in the text. (B) Models of BL pathogenesis. (I) A naïve B cell infected with EBV expresses the latency III programme and is driven to proliferate. EBNA3A and EBNA3C are expressed and this can lead to the epigenetic repression of *Bim*. EBNA2 is down-regulated as the cells to differentiate into germinal centre (GC) cells. They now express only EBNA1, the LMPs and EBER RNAs but transcription of *Bim* is now significantly impaired. *Ig*/*Myc* translocation occurs during somatic hypermutation (SHM) and leads to uncontrolled proliferation. The deregulated *Myc* is potentially lethal, however, the prior repression of *Bim* transcription (and perhaps similar repression of *p14*^*ARF*^ and *p16*^*INK4a*^) allows the cell to survive and proliferate. LMP1 and LMP2A may also repress apoptosis via NF-κB, Ras/PI3K/AKT and ERK/MAPK pathways and EBNA1 may prevent Myc-induced accumulation of p53. Myc-driven proliferation prevents the latently infected cell from becoming a resting memory cell and since the progeny remain in the division cycle, EBNA1 is expressed. The balance between proliferation and apoptosis will be maintained by the epigenetic repression of *Bim* (and perhaps other genes) together with the anti-apoptotic activities of EBNA1. (II) It is possible that during the pathogenesis of BL EBV might infect and rescue a GC B cell that has already sustained an *Ig*/*Myc* translocation. Such a cell may have entered a GC as the result of antigen (Ag) stimulation or some form of non-specific (NS) polyclonal activation as is common in malaria or HIV infections. Since latency III is the likely outcome of EBV infection, multiple survival factors will allow this B-blast-like cell to tolerate deregulation of *Myc*, but precisely how it exits from a germinal centre and how switching to latency I would occur is presently uncertain. Although the sequence of events differs from scenario (I), a latency III-expressing blast will again be the progenitor of BL. (III) In the absence of EBV, activated naïve B cells responding to a specific antigen (Ag) or non-specific (NS) polyclonal activation enter a germinal centre wherein AID is activated to initiate SHM and CRS. Very rarely an *Ig*/*Myc* translocation occurs as the result of aberrant CRS so the apoptosis threshold in this cell and its progeny will be dramatically reduced. For survival the primary targets seem to be p14^ARF^/MDM2/p53 and Myc/Bim, with a strong natural selection for mutations of p53 and/or codon 57/58 mutations of *Myc* (mut-Myc). See text for details and references.

EBV is the γ-herpesvirus associated with eBL and like all γ-herpesviruses is characterised by a tropism for lymphocytes and its ability to persist for the life of the infected host. *In vitro*, EBV can very efficiently induce the activation and continuous proliferation of resting human B cells [57]. This process is known as B cell transformation or immortalization, and the resulting lymphoblastoid cell lines (LCLs) carry the viral genome as extra-chromosomal episomes and express only nine ‘latent’ EBV proteins. There are six nuclear antigens (EBNAs 1, 2, 3A, 3B, 3C and LP) and three membrane-associated proteins (LMP1, LMP2A and 2B). In addition two species of non-coding RNA are expressed (the EBV-encoded RNAs [EBERs] and the BamH1A rightward transcripts [BARTs]). Together these viral factors activate quiescent B cells from G0 into the cell cycle, initiate and sustain proliferation and maintain the viral episome in its extra-chromosomal state (reviewed in [57]). Current data on the asymptomatic life-long persistence of EBV in humans are consistent with the viral genome residing long-term in a resting memory B cell population rather than an LCL-like population of proliferating B-blasts. To establish persistence, EBV infects resting (naïve) B cells and drives these to proliferate transiently as activated B-blasts. This expansion of activated B cells is accompanied by their differentiation to become centroblasts, then centrocytes and finally resting memory B cells (Fig. 2A). Although the precise series of events that the EBV-positive B cells undergo to reach the memory compartment remains uncertain, it occurs in germinal centres [8] and involves the regulated shut-down of latent EBV gene expression from an initial state called latency III (as found in LCLs), via latency II (EBNA1, LMP1, LMP2 and the EBERs) until in quiescent memory B cells no EBV proteins can be detected in a pattern called latency 0. When EBV-positive memory B cells in peripheral blood occasionally re-enter the cell cycle and divide in response to homeostatic triggers, there is transient expression of EBNA1—a state described as latency I (reviewed in [3,58]; Fig. 2A). Central to the growth/proliferation programme (latency III) is the expression of the key EBV transactivator protein EBNA2. This transcription factor is critical for the activation of B cells since EBNA2 mimics notch signalling, induces Myc expression and up-regulates the G1 cyclin D2. Central to latency-state-switching, differentiation and entry into memory is down-regulation of EBNA2 [3,8].

Despite its asymptomatic persistence in the majority of individuals, EBV has a strong association with a number of human cancers of B cell, T cell and epithelial origin [5,59,9]. Since EBV alone is clearly insufficient for malignant transformation, other factors – including the immune status of the infected individual and additional genetic and/or epigenetic changes within the infected cells – must be involved. In addition to BL, EBV is linked to the aetiology of two other important B cell malignancies: Hodgkin's lymphoma and post-transplant lymphoproliferative disease (PTLD). Each of these tumours has a distinct cellular phenotype (or range of phenotypes) and generally expresses a characteristic pattern of EBV latent genes. This suggests that each tumour has a different aetiology and that EBV plays a distinct role in each. However it is likely that all these lymphomas originate from EBV-infected B-blasts derived from resting B cells and that the pattern of EBV gene expression in the tumour reflects that found in the corresponding type of B cell during normal persistence [58].

## 8 EBV latency and apoptosis—continually re-setting the threshold?

During normal, asymptomatic persistence of EBV at least 4 patterns of EBV gene expression are found in B cells—latency III, latency II, latency I and latency 0 (Fig. 2A). These transcription regimes are determined by the usage of specific EBV promoters and probably the differentiation state of the B cell. With the exception of latency 0, which is seen in quiescent memory cells when no EBV proteins are expressed, each of these patterns of expression is recapitulated in an EBV-associated B cell lymphoma: latency III in PTLD/immunoblastic lymphomas, latency II in Hodgkin's lymphoma and latency I in eBL (reviewed in [57,58,60]). At least nine of the EBV latency-associated gene products (summarized in Table 1) have been linked to enhanced cell survival, are capable of re-setting the apoptosis threshold and so might contribute to the aetiology of EBV-positive BL.

### 8.1 EBNA1

EBNA1 is required for the replication and stable persistence of EBV episomes and also transactivates the expression of other EBV latency proteins [57]. Since it is the only EBV protein that is consistently expressed in all EBV-associated cancers, there has been a considerable research effort to determine whether it contributes to oncogenesis, in particular by enhancing cell survival. Good evidence from two independent experimental systems indicates that EBNA1 has anti-apoptotic activities capable of contributing to Myc-associated lymphomagenesis. Using retroviruses to deliver dominant-negative EBNA1 into EBV-positive BL and LCL cells, Sugden and colleagues showed that interfering with EBNA1 activity leads to increased apoptosis. The survival advantage imparted by EBNA1 was not dependent on its ability to maintain EBV episomes, nor whether the cells express a functional, wild type p53 [61]. In contrast, the Frappier group arrived at the conclusion that EBNA1 has an anti-apoptotic activity that is mediated through p53. Using an unbiased approach they identified the ubiquitin-specific protease USP7 (also known as HAUSP) as an EBNA1-interacting protein. USP7 had been shown to bind and de-ubiquitinate p53, leading to reduced proteasome-mediated degradation and p53 stabilization. Since EBNA1 effectively competes with p53 for USP7 binding and indirectly causes reduced p53 stability, it was concluded that EBNA1 would have an anti-apoptotic effect because of its capacity to reduce the level of p53 [62]. Unfortunately no evidence was provided that this occurs in B cells latently infected with EBV and various other studies have shown – at least in LCLs responding to DNA damaging agents – that latent EBV does not reduce the steady state level of p53, block its phosphorylation and accumulation, or impair its function [63,64]. Although the evidence for EBNA1 having anti-apoptotic activity is now compelling, the precise molecular mechanism remains to be determined.

### 8.2 EBNA2

EBNA2, through a physical interaction with Nur77, protects B cells against inducers of apoptosis that affect DNA metabolism, e.g. 5-fluorouracil and etoposide [65,66]. Although Nur77 is a nuclear receptor and transcription factor, it can translocate to mitochondria, bind to Bcl-2, expose a pro-apoptotic BH3 domain on Bcl-2 and so convert it to a death inducer [67]. It has been suggested that the interaction of Nur77 with EBNA2 may help retain Nur77 in the nucleus, however an additional role for this interaction in the modulation of Nur77-regulated transcription cannot be excluded. The function of Nur77 as a transcription factor in the regulation of apoptosis has not been fully explored and neither has the intriguing observation that Nur77 and Bim functionally converge in the elimination of auto-reactive lymphocytes [67,68].

One of the many cellular genes that EBNA2 transactivates – through its mimicry of notch signalling – is *Bfl*-*1*[69]. Bfl-1 (also known as A1) is a close anti-apoptotic relative of Bcl-2 that binds and neutralizes Bim, and has recently been shown to play an important role in the survival of a variety of malignant B cells [70,71]. The EBNA2-mediated induction of *Bfl*-*1* complements its transcriptional induction by LMP1 (see below).

Despite these impressive pro-survival credentials, it is presently unclear whether EBNA2 plays any direct anti-apoptotic role in the pathogenesis of BL unless EBV infects a B cell that has already sustained an *Ig*/*Myc* translocation (see below and Fig. 2B).

### 8.3 EBNA3A and 3C

EBNA3A, EBNA3B and EBNA3C comprise a family of nuclear proteins that probably arose in the evolution of EBV by gene duplication events. Although they are related and share certain features, there is nothing to suggest that they have extensively redundant functions. EBNA3A and EBNA3C are essential for the efficient transformation of B cells, but EBNA3B is dispensable. Both EBNA3A and EBNA3C act as potent repressors of transcription when targeted to DNA and both possess oncogenic activity in primary rodent cells (reviewed in [57,72]).

It has recently been shown that latent EBV can specifically and consistently induce a significant reduction in the expression of Bim [73,74]. This can occur at the level of transcription and Bim protein turnover—the latter probably via LMP2A activation of ERK/MAPK signalling ([73,75] and see below). Recombinant EBV with engineered deletions of specific EBNA genes have demonstrated that EBNA3A and EBNA3C functionally interact to inhibit the expression of Bim in BL cells latently infected with EBV [76]. The turnover of Bim RNA and protein seem to make little or no contribution to this EBV-associated repression, suggesting that transcription of Bim is the primary target in BL and LCLs. Since treatment of EBV-carrying B cells with inhibitors of histone deacetylase and DNA methyltransferase enzymes de-represses *Bim*, this implies that epigenetic mechanisms of transcriptional control are involved in the down-regulation of *Bim*. Consistent with these data, repressive marks on chromatin (e.g. reduced acetylation of histones H3 and H4, trimethylation of lysine 27 on histone H3 and CpG DNA methylation) are found on the *Bim* promoter in EBV-positive BL cells and LCLs but not EBV-negative BL or normal B cells [77]. The precise mechanism by which EBNA3A and EBNA3C mediate repression of *Bim* and the potential involvement of other EBV factors requires further investigation. However, since newly infected normal B cells express the EBV transactivator EBNA2, which constitutively transactivates *Myc*[78] and therefore can indirectly induce Bim expression, EBNA3A and EBNA3C may have evolved to prevent EBNA2-induced, Bim-mediated apoptosis in infected B cells. If they play a role in the development of BL, it is perhaps an unfortunate side effect of this regulatory function that is required to establish EBV latency and persistence. Since *Bim* is a uniquely important tumour suppressor gene in B cells, modulation of its expression by EBNA3A and EBNA3C is likely to be a major contributory factor in the development of any EBV-associated B lymphoma. In the pathogenesis of BL, the data suggest that EBV, through the action of EBNA3A and EBNA3C, is operationally equivalent to codon 57/58 mutants of *Myc*, since in infected cells induction of *Bim* by Myc would be inhibited and Bim levels kept very low. Although EBNA3A and EBNA3C are not generally expressed in GC B cells or eBL, their continued expression may not be required in progeny cells for a profound anti-apoptotic effect to be sustained in the developing tumour as the repression of *Bim* they initiate and/or maintain is epigenetic and therefore heritable. There will be a strong selection pressure to fix the level of *Bim* transcription below a critical threshold.

### 8.4 LMP1 and LMP2

LMP1 is a membrane bound signalling molecule that can drive B cell proliferation by mimicking CD40. It also confers survival advantage on cells via the transcriptional activation of NF-κB-regulated anti-apoptotic genes including *Bcl*-*2*, *A20* and *Bfl*-*1*[57,79–81]. However, LMP1 also up-regulates pro-apoptotic genes involved in B cell proliferation (e.g. *PAC1*, *clarp* and *Myc*). The suggestion is that while stimulating B cell proliferation LMP1-signalling coordinates the level of pro- and anti-apoptotic proteins that is appropriate for the proliferative state of the cell, and thus ensures survival [82].

Like LMP1, LMP2A is a cell membrane-spanning signalling molecule; it mimics a constitutively activated B cell receptor (BCR) and can protect B cells from pro-apoptotic stimuli [83,84]. LMP2A constitutively activates the Ras/PI3K/AKT signalling pathway and this correlates with an increase in the level of anti-apoptotic Bcl-X_L_. Elevation of Bcl-X_L_ expression will neutralize pro-apoptotic Bcl-2-family members, including Bim and Puma. LMP2A also activates the ERK/MAPK pathway [75] and this can lead to phosphorylation of Bim by ERK. This phosphorylation of Bim marks it for ubiquitination and proteasome-mediated degradation. So, taken together these data suggest that LMP2A will very effectively increase B cell survival by two separate pathways targeting both pro-survival and death-inducing factors. Consistent with this, it was recently reported that LMP2A can accelerate Myc-induced lymphomagenesis in a transgenic mouse model that accurately mimics aspects of BL [85,86].

The available data suggest that LMP1 and LMP2A are transiently expressed in GC B cells prior to their differentiation into memory cells and exit from the lymph node. While they are expressed both membrane proteins have the ability to significantly tip the balance of Bcl-2-family members in favour of cell survival. This may be very important when *Myc* is initially deregulated but would not be sustained when their expression is extinguished later in the progression of eBL (see below and Fig. 2B).

### 8.5 BART miRNAs

A family of latent transcripts corresponding to the EBV *Bam*H1A fragment (BARTs) were first described in nasopharyngeal carcinoma (NPC)-derived cells [87]. Despite having potential coding capacity, no protein has been identified as being translated from these multiply spliced transcripts in latently infected B cells (reviewed in [88]). Recently it was demonstrated that EBV is capable of expressing up to 22 micro-RNAs (miRNAs) and that most of these are encoded in the region covered by the BARTs. This cluster of miRNAs (the BART miRs) is robustly expressed in NPC cells, but is either very weakly expressed or absent from EBV-carrying B cells [89]. Furthermore several of the BART miRs are unnecessary for the transformation of B cells, since they lie within a region of the EBV genome that is deleted in the prototype transforming B95.8 strain of virus [89].

One of the few BART miRs to be characterised, BART-mi5, is of particular interest in the context of *Myc* activation because it down-regulates the pro-apoptotic BH3-only protein Puma (p53-up-regulated modulator of apoptosis) [90]. Although BART-mi5 is very abundant in NPC cells, preliminary data suggest it is expressed at almost undetectable levels (or not at all) in B cell lines; however the data on fresh BL biopsy material are not yet in. BART-mi5 is one of the BART miRs deleted from B95.8 EBV and must therefore be dispensable for the *in vitro* transformation of B cells into LCLs. Its importance during the establishment and maintenance of *in vivo* persistence and the pathogenesis of BL remains to be determined.

### 8.6 EBERs

EBERs 1 and 2 are small, non-coding, non-polyadenylated RNAs expressed in all known forms of EBV latency. They are generally very abundant but their mechanism of action in EBV biology and disease pathogenesis remains poorly understood (reviewed in [88]). Several claims have been made about the anti-apoptotic effects of the EBERs, however these are somewhat controversial. The current consensus of opinion is that the EBERs may furnish B cells with a degree of protection from the pro-apoptotic activity of α-interferon, but the molecular basis for this and significance in BL are unknown [88].

### 8.7 BHRF1 and BALF1

BHRF1 is an EBV Bcl-2 homologue (a vBcl-2) that binds and inactivates Bax, directly inhibiting the mitochondrial apoptotic machinery [91]; unsurprisingly over-expression very effectively suppresses apoptosis, including Myc-induced apoptosis [92,93]. BALF1 is also a vBcl-2, but its role in the suppression of apoptosis is more controversial—it is not clear whether it has anti-apoptotic activity under all circumstances [94]. Because both BALF1 and BHRF1 are expressed during the lytic replication cycle of EBV and the proteins are generally undetectable in strictly latent B cells, it was assumed they play no role in latency. However by employing recombinant EBV-carrying specific gene deletions, Altmann and Hammerschmidt revealed a rather surprising transient burst of BHRF1 and BALF1 expression early after the infection of explanted resting B cells that is necessary for their transformation into LCLs. Protection from apoptosis in newly infected B-blasts therefore appears to be biphasic: initially BHRF1 (and probably BALF1) bind to and directly inactivate pre-existing pro-apoptotic Bcl-2-family members and provide immediate protection, they are then superseded by the truly ‘latent’ proteins acting in a variety of ways a day or two later (Fig. 2A and Table 1). The molecular mechanism for this rapid, transient activation of BHRF1 and BALF1 and the subsequent switch to regular type III latency remains to be determined [95]. As with EBNA2, this early surge of anti-apoptotic activity could play an important role in eBL if EBV infects a cell with a pre-existing *Ig*/*Myc* translocation.

## 9 EBV and senescence

It is unclear whether B cells can respond to oncogenic stress by cell cycle arrest and senescence—the default pathway may be terminal differentiation (for more detailed discussion of senescence and differentiation pathways in B cells see [72,96]). Nevertheless, at least three EBV latent proteins have the ability to inhibit senescence in fibroblasts exposed to an activated oncogene: EBNA3C, EBNA3A and LMP1 all appear to rescue fibroblasts from arrest induced by oncogenic Ras and this probably involves repression of the gene encoding p16^INK4a^.

### 9.1 EBNA3C

EBNA3C can behave like adenovirus E1A and papillomavirus 16/18 E7 proteins in oncogene cooperation assays using primary rat embryo fibroblasts, REFs. That is, it can cooperate with oncogenic Ras (Ha-Ras) in the transformation and immortalization of REFs [97]. Expressing Ha-Ras alone in these cells induces arrest and premature senescence associated with the induction of G1 regulators p19/14^ARF^, p53 and p16^INK4a^ (reviewed in [37]). This indicated that EBNA3C might target the *CDKN2A* locus encoding both p14/19^ARF^ and p16^INK4a^. Subsequently, using an EBV conditional for EBNA3C function made by fusing EBNA3C with a modified oestrogen receptor, Maruo and colleagues showed that EBNA3C does indeed repress expression of *p16*^*INK4a*^ and *p14*^*ARF*^ in LCLs. Removing the inducer of EBNA3C activity (4-hydroxytamoxifen) from the growth medium results in reduced proliferation and an accumulation of mRNA corresponding to *CDKN2A* and both p16^INK4a^ and p14^ARF^ proteins ([98]; S. Maruo, personal communication and our unpublished observations). By targeting the *CDKN2A* locus, in theory EBNA3C can mimic Bmi-1, inhibit Myc-induced senescence and apoptosis, and because in cycling cells *CDKN2A* is generally repressed by epigenetic modification of histones [25], there could be selection for this reduced expression and eventually promoter DNA methylation.

### 9.2 EBNA3A

EBNA3A also cooperates with Ha-Ras in the transformation and immortalization of REFs [99]. Furthermore, a 4-hydroxytamoxifen-dependent LCL conditional for EBNA3A function showed that in the absence of EBNA3A, cell proliferation gradually grinds to a halt. However, although it was not reported whether this involved regulation of the *CDKN2A* locus [100], it has recently been reported that repression of *p16*^*INK4a*^ transcription in LCLs is associated with EBNA3A expression [101]. Therefore, as with the regulation of *Bim*, there is evidence that EBNA3A and EBNA3C functionally interact. This is consistent with the observation that both proteins suppress Ras-induced senescence in oncogene cooperation experiments [97,99].

### 9.3 LMP1

LMP1 can inhibit the premature senescence induced in mouse embryo fibroblasts (MEFs) by oncogenic Ras and in the process prevents the accumulation of p16^INK4a^[102]. Similar results were obtained in IMR90 pre-senescent diploid human fibroblasts. This LMP1-mediated inhibition of *p16*^*INK4a*^ transcription was reported to involve the redistribution of the Ets-2 transactivator of *CDKN2A*, from nucleus to cytoplasm [103]. Although all these experiments were performed using fibroblasts, it is reasonable to speculate that LMP1 may also transiently contribute to the repression of *CDKN2A* in B cells.

## 10 Does latent EBV create an environment in which an *Ig*/*Myc* translocation can be tolerated?

From a consideration of the preceding sections, it is obvious that the simple answer to this question is yes—during latency *in vivo* EBV can express a substantial arsenal of survival factors that would suppress Myc-driven apoptosis and senescence and favour Myc-driven proliferation. The more pertinent questions are which viral genes are involved and in what sequence are they expressed in the pathogenesis of BL? In the past there was considerable debate about the type of B cell in which the *Ig*/*Myc* translocation occurs and what came first—virus or translocation? (reviewed in [11]). Nevertheless, there is now a growing consensus that the translocation happens in a GC B cell when AID activity is high and that EBV must infect either a naïve B cell, prior to differentiation, or an activated GC B cell. In the latter case infection could be before or after the gene translocation event. Three possible scenarios are considered – two involving EBV, and for comparison the pathogenesis of EBV-negative BL (Fig. 2B). The first model for EBV-positive BL fits best with our current knowledge of EBV biology and the molecular events in BL pathogenesis, but a second alternative pathway is also considered.

### 10.1 An EBV-infected naïve B cell as the progenitor of eBL

A naïve B cell that is infected with EBV in the tonsil expresses the latency III programme and is driven to proliferate in part by EBNA2 activation of *Myc*. EBNA3A and EBNA3C are also expressed and this can lead to the epigenetic repression of *Bim*. EBNA2 expression is extinguished allowing the cells to differentiate into germinal centre (GC) cells. They now express only EBNA1, LMP1, LMP2A and EBER RNAs (it is not known whether the BART RNAs are expressed), however the transcription of *Bim* may still be significantly impaired. *Ig*/*Myc* translocation occurs at this GC stage during SHM and leads to uncontrolled proliferation. Because LMP1 can induce *AID* transcription [104] this might increase the chances of aberrant or sustained AID expression and the likelihood of a translocation event. The deregulated *Myc* is potentially lethal, since normally it would induce apoptosis via the p14^ARF^/MDM2/p53 axis and Bim. However, the prior repression of *Bim* transcription (and perhaps similar repression of *p14*^*ARF*^ and *p16*^*INK4a*^) allows the cell to survive and proliferate. At this stage LMP1 and LMP2A will also repress apoptosis via NF-κB, Ras/PI3K/AKT and ERK/MAPK pathways and EBNA1 may prevent Myc-induced accumulation of p53. Myc-driven proliferation prevents the latently infected cell from becoming a resting memory cell. Since the progeny cells remain in the division cycle and the EBNA1 promoter used in BL cells is E2F-responsive [105,106], so EBNA1 is expressed in the growing BL. The balance between proliferation and apoptosis will be maintained by the epigenetic repression of *Bim* (and perhaps other genes) together with the anti-apoptotic activities of EBNA1. Since both Myc and EBV may enhance genomic instability (see later) then other epigenetic and/or genetic changes that enhance tumour progression (e.g. resistance to damaged DNA or hypoxia) will rapidly ensue and be fixed by further natural selection.

### 10.2 EBV infects a GC B cell

EBV normally infects resting naïve B cells, but it is possible that during the pathogenesis of BL it infects and rescues a GC (or post-GC) B cell that is undergoing or has already sustained an *Ig*/*Myc* translocation. Such a cell may have entered a GC as the result of antigen stimulation or some form of non-specific polyclonal activation as is common in both malaria and HIV infections. All the available data suggest that EBNA2-dependent latency III is the resulting pattern of EBV gene expression when GC and post-GC B cells are directly infected *ex vivo*[107–110]. Although we cannot be certain the outcome of infecting a cycling GC cell *in vivo* will be the same, it is likely that the full arsenal of EBV-encoded survival factors would initially allow B-blast-like cells to tolerate deregulation of *Myc*. However this scenario is problematic since it is unclear how these EBNA2-positive cells would exit from the germinal centre and – most importantly – how they would switch from latency III to latency I. Although the sequence of events differs from scenario (i), the assumption is that a latency III-expressing blast will still be the progenitor of BL and again any epigenetic changes (e.g. repression of *Bim*) and the anti-apoptotic activities of EBNA1 will ensure the survival and proliferation of the latency I-expressing progeny cells. For a more detailed and informed discussion of the behaviour of EBV-infected GC B cells, see reference [8].

### 10.3 EBV-negative sBL

In the absence of EBV, activated naïve B cells, responding to a specific antigen or non-specific polyclonal activation, will migrate to a germinal centre wherein AID is activated to initiate SHM and CRS. In this case aberrant CRS (rather than SHM) may result in *Ig*/*Myc* translocation. The apoptosis threshold in this cell and its immediate progeny will be dramatically reduced and they will be eliminated unless selection favours cells in which the balance between apoptotic and anti-apoptotic factors is tipped in favour of survival. Primary targets seem to be p14^ARF^/MDM2/p53 and Myc/Bim, with a strong selection for pre-existing or rapidly generated mutations of p53 and/or codon 57/58 mutations of *Myc* that could also result from aberrant AID activity. However, the risk of the second oncogenic lesion occurring in the same cell will be extremely low, no doubt accounting for the general rarity of sBL. As with EBV-positive BL, tumour development will select further genetic and epigenetic changes that help evade further stresses such as hypoxia or DNA damage.

## 11 Myc, EBV and genomic instability

Genomic instability (GI) is a hallmark of many cancers. This is probably because ongoing mutations associated with GI increase the frequency of oncogenic changes that feed natural selection during tumour progression.

In addition to its directly transforming role, evidence has been accumulating for several years that deregulated Myc can also produce GI. By the induction of genotoxic reactive oxygen species (ROS), Myc can evoke double-stranded DNA breaks and chromosomal aberrations that in turn can initiate apoptosis via the Atm/Chk2/p53 DNA damage response (DDR) pathway [111–113]. As a consequence the formation of Myc-driven lymphomas may rely on suppression of not only the ARF/MDM2/p53 and Bim tumour-suppressor pathways, but also the Atm/Chk2/p53 cascade. Furthermore a very recent study has revealed that the aberrant control of protein synthesis in *Eμ-Myc* lymphomas is also responsible for supernumerary centrosomes, cytokinesis defects, GI and aneuploidy [28]. This means that survival of Myc-driven lymphoma may in addition require suppression of mitotic checkpoints and/or the pathways leading to mitotic catastrophe [114].

Coincidentally, data have been accumulating that indicate latent EBV also induces GI [115,116] and this has culminated with the discovery by the Masucci group that EBNA1 expression in tumour-derived B cells promotes GI via the transcriptional activation of *Nox2* and induction of ROS [117]. If indeed EBNA1 generates ROS during normal EBV latency, and as a result induces GI, then it is conceivable that EBV has evolved suppressors of cell cycle checkpoints and/or modifiers of the DDR to ensure that there are no delays or blocks to proliferation during the establishment of persistence. Although the data remain very sketchy, several lines of investigation suggest that the EBNA3 proteins (in particular EBNA3C) may occupy this role by inhibiting G2 and/or mitotic checkpoints ([74,118–121] reviewed in [72]).

Since Myc and EBNA1 independently induce ROS and GI, one cannot exclude the possibility that in the pathogenesis of BL their effects are additive and that, as Masucci and colleagues suggest, this may be further enhanced by the expression of other viral proteins – such as EBNA3C – suppressing checkpoints [117]. The resulting ability to survive and proliferate with severely damaged DNA could account for the unexpected chromosomal complexity recently revealed in BL by spectral karyotyping and comparative genomic hybridization [122]. Furthermore, it may help to explain the significance of the subset of BL that retain expression of the EBNA3 proteins (also known as Wp-restricted BL [60,123,124]). Since ROS will induce DNA damage capable of activating the ATM/Chk2/p53 cascade this could be why classical latency I eBL often develop mutations in p53, whereas the Wp-restricted BL lines – because they express EBNA3s that can inhibit G2 and M checkpoints – are able to retain wild type p53 (see for example [76]).

## 12 Concluding remarks

EBV is clearly a major risk factor for most BL, and it seems likely that this is because EBV complements the activation of *Myc* by suppressing the ability of deregulated Myc to induce apoptosis and possibly cell cycle arrest. This can result from the direct action of EBV gene products on apoptosis and/or senescence pathways at critical points in the development of BL. However, there is also the fascinating prospect that by initiating epigenetic changes in the host genome (e.g. *Bim* and perhaps *CDKN2A* or other genes) EBV may re-programme B cells so that their progeny survive oncogenic stress and genomic instability and are more likely to become cancerous. It is now just over 50 years since BL was described by Burkitt, about 45 years since its aetiology was linked with EBV and about 25 years since it was first appreciated that chromosomal translocation of *Myc* to an *Ig* locus (rather than infection with EBV) is the defining characteristic of the tumour. As we learn more about Myc and the consequences of its deregulation, so it seems we understand more about the role of EBV in BL. Hopefully this conceptual framework and these complementary lines of research will gradually lead to a more complete understanding of this remarkable tumour.

## Conflicting interest statement

The author declares that there is no conflict of interest.

## Figures and Tables

**Table 1:** EBV latency gene products^a^ that can suppress apoptosis and/or senescence.

EBV gene product	Targets	Outcomes/comments	References
EBNA1	USP7	Reduces p53 stability	[62]
		Anti-apoptotic	
		Anti-senescence?	
	Unknown	Anti-apoptotic	[61]

EBNA2	Nur77	Nuclear retention of Nu77	[65,66]
		Anti-apoptotic	
	*Bfl-1*	Increases expression of Bfl-1	[69]
		Anti-apoptotic	

EBNA3A	*Bim*	With EBNA3C, represses *Bim* expression	[76,77]
		Anti-apoptotic	
	*CDKN2A* (*p16*^*INK*4*a*^)	Represses *p16*^*INK*4^ expression	[101]
		Anti-senescence	

EBNA3C	*Bim*	With EBNA3A, represses *Bim* expression	[76,77]
		Anti-apoptotic	
	*CDKN2A* (*p16*^*INK*4*a*^ and *p14*^*ARF*^)	Represses *p16*^*INK*4*a*^ and *p14*^*ARF*^ expression	[98]; S. Maruo, personal communication; our unpublished data
		Anti-senescence	
		Anti-apoptotic?	

LMP-1	*Bcl-2*, *Bfl-1*, *A20*	Via NF-κB signalling, increases expression of Bcl-2, Bfl-1, A20	[79–81]
		Anti-apoptotic	
	*p16*^*INK*4*a*^	Reduces expression of p16^INK4a^	[102,103]
		Anti-senescence	

LMP-2A	*Bcl-x*_*L*_	Via Ras/PI3K/AKT can increase Bcl-x_L_ expression	[83–85]
		Can cooperate with Myc in lymphomagenesis	
		Anti-apoptotic	
	Bim	Via ERK/MAPK may induce degradation of Bim	[73,75]
		Anti-apoptotic	

miR-BART5	*Puma mRNA*	Reduces Puma expression	[90]
		Anti-apoptotic	

EBERs	Unknown	Anti-apoptotic	[88]

BHRF1^a^	Bax/Bak	Binds to and inhibits Bax/Bak	[91–93,95]
		Suppresses Myc-induced apoptosis	
		Anti-apoptotic	

a It is currently unclear whether BHRF1 is a *bona fide* latent gene product or whether it is only transiently expressed immediately after infection then again on entry to the lytic cycle [95].
